# Effect of Microstructure Refinement on Surface Morphology and Dynamic Mechanical Properties of W-Cu Alloys

**DOI:** 10.3390/ma14164615

**Published:** 2021-08-17

**Authors:** Fei Ding, Jinglian Fan, Liqiang Cao, Qidong Wang, Jun Li, Pengfei Li

**Affiliations:** 1 State Key Laboratory of Powder Metallurgy, Central South University, Changsha 410083, China; lip166@163.com; 2 Institute of Microelectronics of Chinese Academy of Sciences, Beijing 100029, China; caoliqiang@ime.ac.cn (L.C.); wangqidong@ime.ac.cn (Q.W.); lijun@ime.ac.cn (J.L.)

**Keywords:** W-Cu alloy, ultrafine-grained, coarse-grained, coating, dynamic impact

## Abstract

Two ultrafine-grained W-Cu alloys and two coarse-grained W-Cu alloys were prepared to study the effect of tungsten grain refinement on the interface characteristics between coating and W-Cu alloys. The experimental results show that in the coarse-grained W-Cu alloys, the tungsten phase near the surface is easy to form pits and cracks during impact loading, while the fine-grained tungsten alloy is not prone to this phenomenon. Simulations show that refining tungsten grains can not only reduce the impact absorption energy, but also increase the contact area of tungsten and copper phases, thereby reducing the fracture probability of tungsten phases and improving the dynamic mechanical properties of the W-Cu alloys under impact loading. The tested results show that the shear strength of gold studs on the coating is increased by about 33%, after grain refinement for the W-Cu alloys.

## 1. Introduction

Tungsten (W)–copper (Cu) composite combines the excellent physical properties of W and Cu. The rigid skeleton structure of W provides good mechanical properties and low thermal expansion coefficient, while the network structure of Cu offers excellent thermal conductivity and electrical conductivity [1,2,3]. Accordingly, W-Cu alloys have attracted much attention in electrical packages and integrated-circuit heat-sinks [4,5]. The infiltration of porous-sintered tungsten skeleton by liquid copper is one of the most common methods to form W–Cu composites [6,7,8]. Other methods used to fabricate W-Cu composites include thermo-mechanical process [9,10,11], spark plasma sintering [12], and liquid phase sintering [13,14,15]. Refinement of W grains is an effective way to improve mechanical properties of the W-Cu alloys [16,17,18,19].

In order to meet the requirements of electrical packages, W-Cu alloys need to be welded with other dissimilar materials. The surface of W-Cu alloys is easily oxidized, leading to poor weldability. In order to improve the welding performance, it is necessary to plate coating on the surface. Prior to coating, the W-Cu alloy needs to be sandblasted to remove the oxide layer, the purpose of which is to increase the surface roughness of the substrate and enhance the interfacial bonding between the coating and the substrate.

Therefore, the impact on the surface of W-Cu alloy plays a decisive role in the surface morphology of W-Cu alloy and the adhesion of coating and substrate. Gao et al. [20] studied the influence of surface nano-crystallization on the surface morphology and microstructure of W-Cu alloy by surface peening to explore the grain refinement mechanism of supersonic fine particles bombarding (SFPB). However, the research on fine-grained W-Cu alloys mainly focuses on the microstructure characteristics of the surface layer, and the research on its dynamic mechanical properties at high strain rates is very limited.

Andrews et al. [21] used the conical indentation analysis model of an elastic-plastic solid to calculate the maximum indentation depth, coefficient of restitution, and contact time for various metals and indentation velocities. However, the former study was focused on the averaged dynamic response rather than the transient dynamic response. Lee et al. [22] extended the dynamic indentation analysis of fully elastic-plastic solids to strain-hardened solids, showing strain-rate-independent or strain-rate-dependent deformation. Although important insights into the indentation mechanics of elastic-plastic materials have been obtained from the above researches and other similar works, the shock deformation has significant implications in energy absorption. Tan et al. [23] emphasized the meso-structural reasons for the enhanced energy absorption impact and based on their compression simulations of honeycombs with a rate-independent base material, they proved that energy absorption increased significantly with impact velocity. Accordingly, there is very limited information concerning the influence of the microstructure of W-Cu alloy on its surface dynamic mechanical properties under impact load. To address this issue, in this work, two ultrafine-grained W-Cu alloys and two coarse-grained W-Cu alloys were prepared. The effects of grain refinement on the surface morphology and dynamic mechanical properties of the W-Cu alloys under impact loading were studied.

## 2. Materials and Methods

For the preparation of ultrafine-grained 80W-20Cu alloy (UFG WCu20) and ultrafine-grained 50W-50Cu alloy (UFG WCu50), W-20 wt.% Cu and W-50 wt.% Cu nanocrystalline composite powders were prepared by sol-spray drying and a subsequent hydrogen reduction process, followed by sintering at temperatures between 1150 and 1260 °C for 90 min. More details on the production route and microstructure characterization of the powders can be found in our previous work [24].

For the preparation of coarse-grained 80W-20Cu alloy (CG WCu20) and rolling-deformed coarse-grained 80W-20Cu alloy (RD-CG WCu20), W powder (purity ≥ 99.9 wt.%, with an average particle size of 5 μm) was first isostatic cold pressed into W skeleton under a pressure of 400 MPa. Then, the W skeleton was sintered at 1100 °C for 2 h. The sintered W skeleton was infiltrated with pure copper at 1350 °C in a hydrogen atmosphere for 90 min. The obtained CG WCu20 alloy was rolled at 800 °C and heat-treated at 800 °C in nitrogen atmosphere, repeated three times until a deformation amount of 80% was achieved.

Prior to coating, alumina abrasive particles with a mean size of 100 μm were used to impact the surface of the four W-Cu alloys at an impact velocity of 15 m/s (spray gun pressure 0.5 MPa) and the impact time was 1 min. Coating was then performed by the electroless deposition of nickel (2–4 μm) and gold (0.1–0.5 μm) on the surface of W-Cu alloys. The microstructure characterization was performed using scanning electron microscopy (Zeiss Sigma HD, Carl-Zeiss, Oberkochen, Germany).

In order to evaluate the bonding performance of the coating, a shear test was performed using a gold stud which is scraped from the metalized connection supports. The gold studs were bonded to the coating of W-Cu alloys by ultrasonic welding with gold wires. The shear test equipment used in this work was TRY MFM1500, with a constant movement speed at about 100 μm/s.

## 3. Results and Discussions

### 3.1. Microstructure Characteristics of W-Cu Alloys

**Figure 1:**
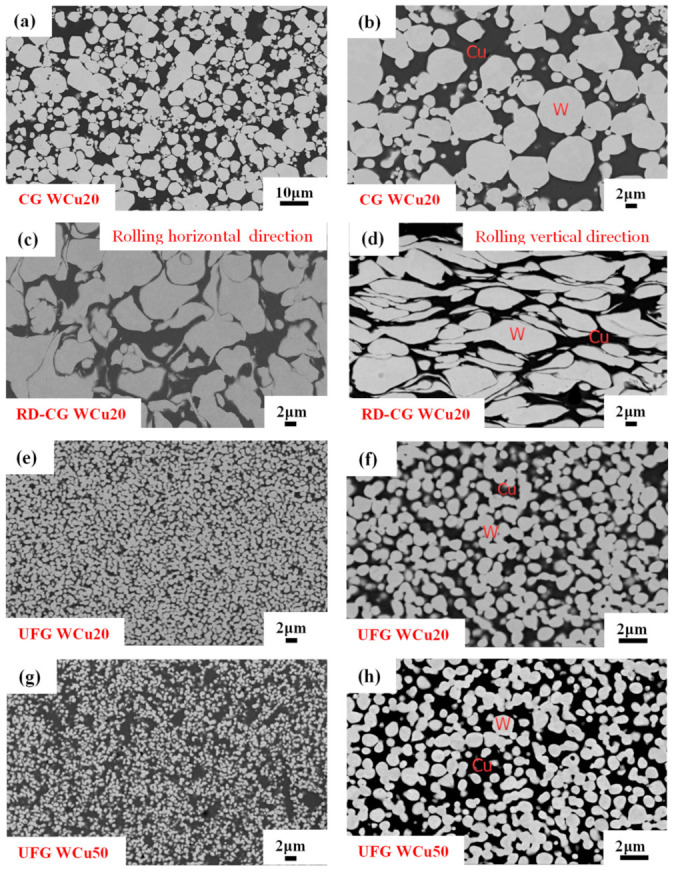
Microstructures of CG WCu20 (**a**,**b**); RD-CG WCu20 (**c**,**d**); UFG WCu20 (**e**,**f**); UFG WCu50 alloys (**g**,**h**).

Figure 1 shows the microstructures of CG WCu20, RD-CG WCu20, UFG WCu20, and UFG WCu50 alloys, in which the gray white particles are W phase and the black ones are Cu phase. In addition, the interface between the W and Cu appears tight, without major defects. The W phase of the CG WCu20 (Figure 1a,b) has a certain agglomeration, and the size distribution varies from 2 to 10 μm. After the W-Cu alloy is deformed by rolling (Figure 1c,d), the tungsten particles are mostly elongated along the rolling horizontal direction, and the maximum length of the elongated tungsten grains is more than 20 μm. In contrast, in the UFG WCu20 and UFG WCu50 alloys (Figure 1e–h), the W phase is homogeneous, and no obvious Cu or W agglomeration can be observed, and the average grain size of W is below 1 μm. Moreover, the tungsten grains in UFG WCu50 are smaller than those in the UFG WCu20 alloy.

### 3.2. Surface Morphologies of W-Cu Alloys

**Figure 2:**
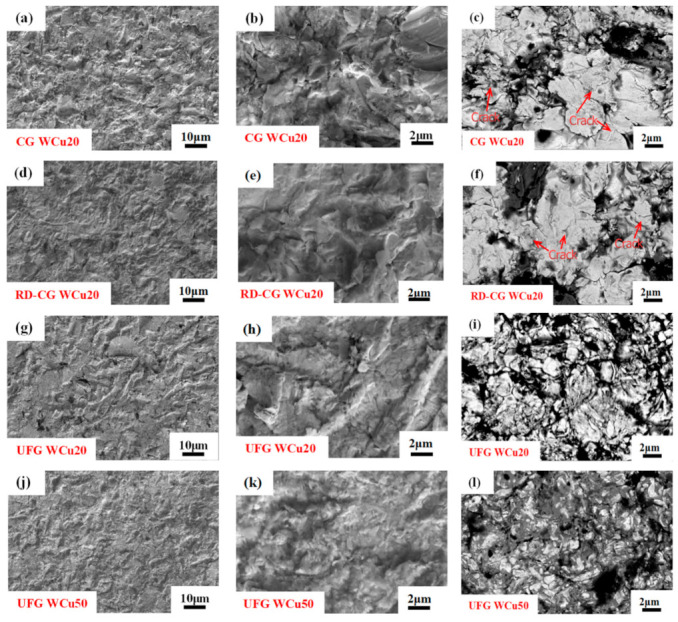
The surface morphologies of CG WCu20 (**a**–**c**); RD-CG WCu20 (**d**–**f**); UFG WCu20 (**g**–**i**) and UFG WCu50 alloys (**j**–**l**) after sandblasting.

**Figure 3:**
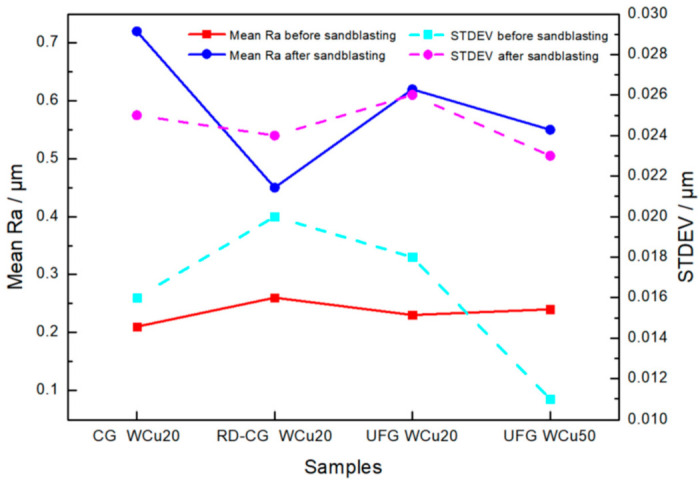
Roughness of the four W-Cu alloys before and after sandblasting.

Figure 2 shows the surface morphologies of CG WCu20, RD-CG WCu20, UFG WCu20, and UFG WCu50 alloys after sandblasting. It can be seen from Figure 2a–f that after sandblasting, the tungsten phase (the white contrast) on the surface of the CG WCu20 and RD-CG WCu20 alloys aggregates and exhibits a flat structure with many visible cracks. By contrast, in the UFG WCu20 (Figure 2i,l), the W phase also shows obvious aggregation on the surface and exhibits a flat structure, but there are much less cracks on the tungsten phase. Meanwhile, due to the high Cu content in UFG WCu50 alloy, a large amount of Cu (the dark contrast) is attached to the surface of the W phase, and no cracks can be seen on the W phase. Figure 3 compares the surface roughness of the four W-Cu alloys before and after the sandblasting. It can be seen that, after sandblasting, the surface roughness has increased significantly, and it is beneficial to enhance the interfacial bonding between the coating and the substrate.

### 3.3. Interface Characteristics between the Coating and W-Cu Alloys

**Figure 4:**
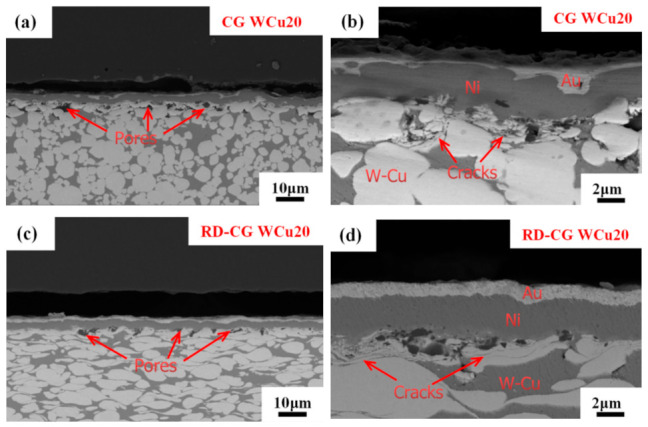

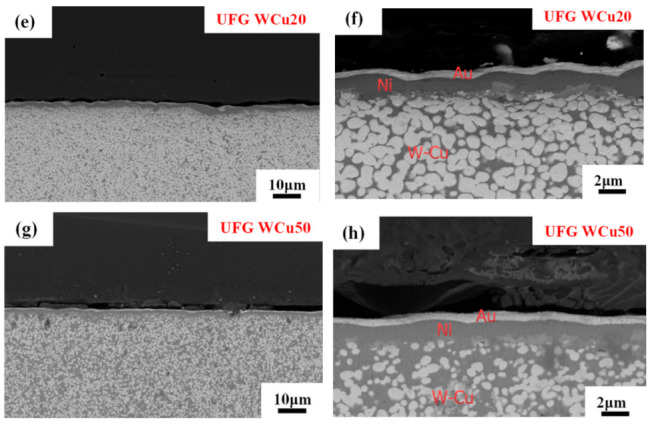
Cross-sectional microstructures of CG WCu20 (**a**,**b**); RD-CG WCu20 (**c**,**d**); UFG WCu20 (**e**,**f**) and UFG WCu50 (**g**,**h**) alloys after coating.

Figure 4 shows the cross-sectional microstructure of the coating on the surface of the W-Cu alloys. Clear differences in the bonding interfaces between the four W-Cu alloys and the coating metals (i.e., Ni and Au) can be seen. In the case of the CG WCu20 and RD-CG WCu20 alloys (see the red arrows in Figure 4a,c), a large number of pores are visible at the interfaces between the coating metal layer and the substrate. The surface of these two W-Cu alloys is relatively loose, and tungsten grains are delaminated at the pores (see the red arrows in Figure 4b,d). By contrast, the UFG WCu20 and UFG WCu50 alloys (Figure 4e–h) show much denser interfaces and no evident pores exist, indicating good interfacial bonding between the coating and the matrix of these two W-Cu alloys.

### 3.4. Simulation Analyses of Dynamic Mechanical Behavior during Surface Impact

**Figure 5:**
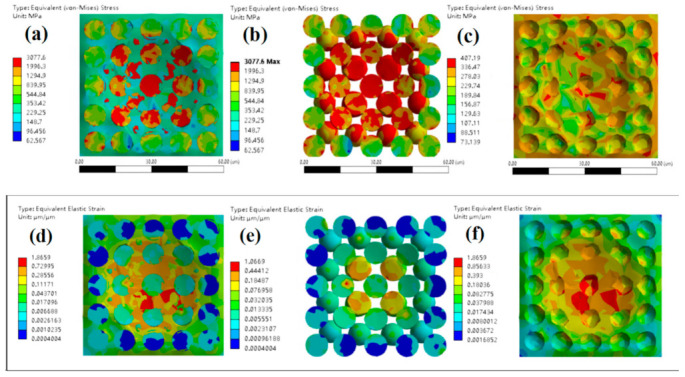
The surface impact dynamic mechanics simulation results of CG WCu20 at an impact velocity of 15 m/s at 25 °C. (**a**) equivalent (von-Mises) stress of CG WCu20; (**b**) equivalent (von-Mises) stress of the W of CG WCu20; (**c**) equivalent (von-Mises) stress of the Cu of CG WCu20; (**d**) equivalent elastic strain of CG WCu20; (**e**) equivalent elastic strain of the W of CG WCu20; (**f**) equivalent elastic strain of the Cu of CG WCu20.

**Figure 6:**
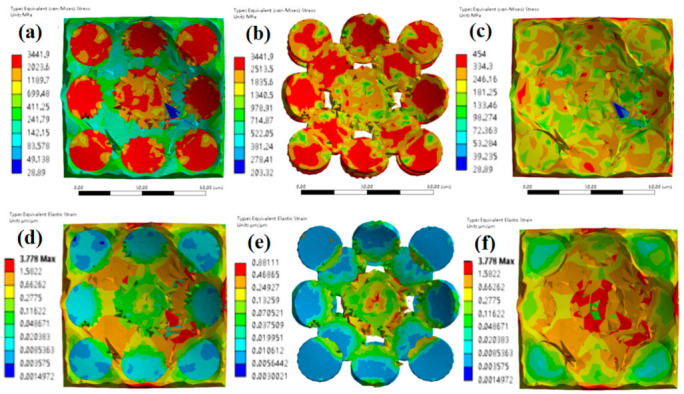
The surface impact dynamic mechanics simulation results of RD-CG WCu20 at an impact velocity of 15 m/s at 25 °C. (**a**) equivalent (von-Mises) stress of RD-CG WCu20; (**b**) equivalent (von-Mises) stress of the W of RD-CG WCu20; (**c**) equivalent (von-Mises) stress of the Cu of RD-CG WCu20; (**d**) equivalent elastic strain of RD-CG WCu20; (**e**) equivalent elastic strain of the W of RD-CG WCu20; (**f**) equivalent elastic strain of the Cu of RD-CG WCu20.

**Figure 7:**
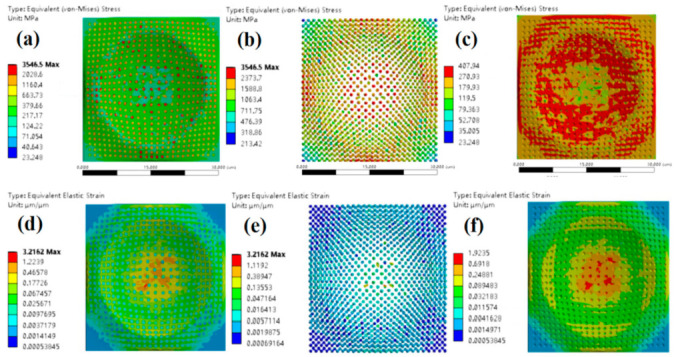
The surface impact dynamic mechanics simulation results of FG WCu20 at an impact velocity of 15 m/s at 25 °C. (**a**) equivalent (von-Mises) stress of FG WCu20; (**b**) equivalent (von-Mises) stress of the W of FG WCu20; (**c**) equivalent (von-Mises) stress of the Cu of FG WCu20; (**d**) equivalent elastic strain of FG WCu20; (**e**) equivalent elastic strain of the W of FG WCu20; (**f**) equivalent elastic strain of the Cu of FG WCu20.

**Figure 8:**
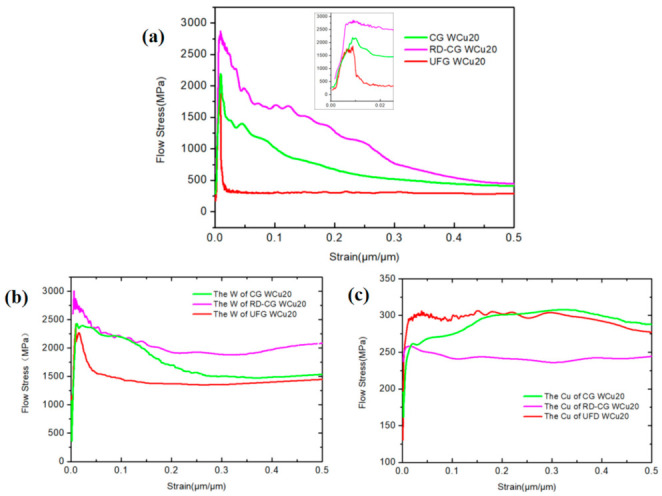
The flow stress–strain simulation curves of three W-Cu alloys under dynamic impact. (**a**) the flow stress–strain simulation curves of CG WCu20, RD-CG WCu20 and UFG WCu20; (**b**) the flow stress–strain simulation curves of tungsten in the CG WCu20, RD-CG WCu20 and UFG WCu20; (**c**) the flow stress–strain simulation curves of copper in the CG WCu20, RD-CG WCu20 and UFG WCu20.

Simulation analyses on the dynamic mechanical behavior of the W-Cu alloys during the surface impact process were conducted by ANSYS finite element analysis (FEA) to study the influence of tungsten grain refinement. The Johnson–Cook model [25] was employed for the simulation and the model constants can be found in Table 1. It can be seen from Figure 5, Figure 6 and Figure 7 that, for CG WCu20 and RD-CG WCu20 alloys, the corresponding stress and strain of tungsten are mainly concentrated in the center of the impact contact. While in UFG WCu20, the stress and strain distribution of the tungsten phase is relatively uniform. Figure 8 shows the simulated flow stress–strain curves of three W-Cu alloys under dynamic impact.

An energy absorption diagram is obtained by plotting the absorbed energy of W as a function of the stress. The absorbed energy is the area under the stress–strain curve, which can be calculated using the following formula [27]

(1) $$W=\int_{0}^{\varepsilon }\sigma (e)\mathrm{de}$$

where σ and ε are the nominal compressive stress and strain, respectively.

As shown in Figure 8a, according to Equation (1), the RD-CG WCu20 absorbs the most energy, while the UFG WCu20 absorbs the least. The structure of W-Cu alloy is composed of hard W phase and soft Cu binder phase. The structural characteristics of W-Cu alloy with two-phase composite material determine the sensitivity of its mechanical properties to the microstructure. Under the same impact velocity and impact carrier, since tungsten is brittle, the increase in the impact area of the tungsten phase leads to a decrease in the impact strain rate, an increase in the yield stress and flow stress, a reduction in the plastic deformation as well as relatively brittle fracture of this material.

**Figure 9:**
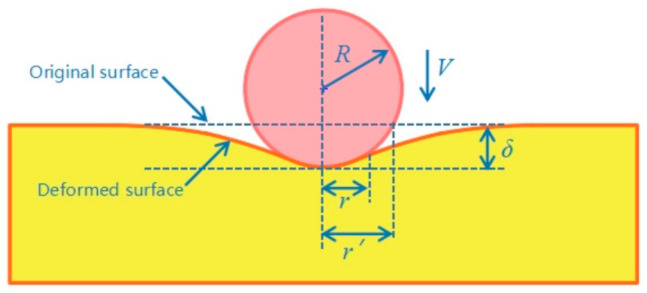
Schematic illustration of spherical indentation with relevant nomenclature.

Figure 9 shows a rigid sphere with a radius R that moves at a speed V and retracts the deformable half space by a depth δ. The resulting contact radius is represented by r, and $r\prime ={\left(2R\delta -\delta^{2}\right)}^{1/2}$ [28] is the corresponding truncated contact radius. The dimensionless indentation depth is represented by δ/r″, where contact is confined to discrete microcontacts defined by the truncation of presumed rigid rough surface by the deformable countersurface. Table 2 shows the simulation result δ and the calculation δ/r′ for the three W-Cu alloys, the dynamic contact area differs significantly from that corresponding to the three W-Cu alloys, especially for the UFG WCu20 alloy (δ/r′ = 0.02). During the impact process of the W-Cu alloy surface, the contact area between the tungsten grains and alumina sand particles directly affect the stress and strain response. The tungsten grains on the surface are flat in the RD-CG WCu20 alloy, and the impact strain energy is absorbed mostly by the tungsten phase. Therefore, the tungsten phase has the highest probability of fracture for the RD-CG WCu20 alloy.

**Figure 10:**
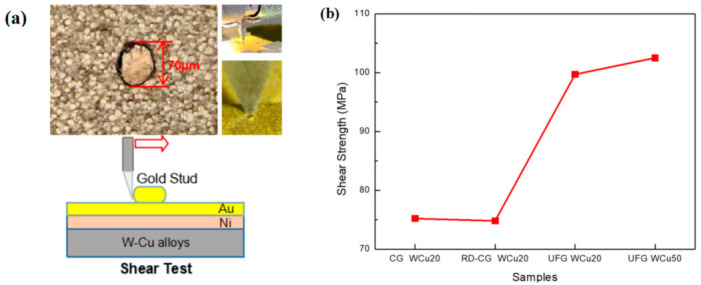
The measured shear strength of gold stud (mean diameter 70 μm) on the Au/Ni coating for the three W-Cu alloys. (**a**) shear strength measured diagrams and test method schematic diagram; (**b**) shear strength test values of different WCu alloys.

According to the principle of constant volume, in a W-Cu alloy with the same tungsten content and the tungsten grains are considered ideal spherical, the diameter of spherical tungsten grains is reduced by 10 to 20 times, and its specific surface area will increase by 10 to 20 times accordingly. If the existence of W-W bonding interfaces is not considered, the bonding interfaces of W and Cu will increase by 10 to 20 times. The tungsten grains in the fine-grained W-Cu alloy are more evenly distributed in the copper matrix, and the grain refinement increases the bonding interface of tungsten and copper. When the W-Cu surface is subjected to dynamic impact, the impact energy of the tungsten grains in the fine-grained W-Cu alloy will be better transferred to the copper substrate and refining the tungsten grains can strengthen the copper matrix (Figure 8c). As a result, the tungsten phase has the lowest probability of fracture for the UFG WCu20 alloy compared with CG WCu20 and RD-CG WCu20. With the decrease of tungsten content in the UFG WCu alloy, the probability of tungsten grain fracture is further reduced, as shown by UFG WCu50 (Figure 4h). Figure 10 shows the measured shear strength of gold stud (mean diameter 70 μm) on the Au/Ni coating for the three W-Cu alloys. The results show that after grain refinement, the shear strength of gold studs is increased by about 33%, and it increases as the tungsten content decreases. The reason is that the interfacial bonding between the coating layer and the tungsten copper surface is improved through grain refinement, and the existence of interface voids is eliminated. It indicates that the density and uniformity of the coating layer are improved, which is beneficial to bond the gold studs and the coating during the ultrasonic bonding process. In addition, other studies [29] have shown that when the diameter of gold studs is 110 μm, the values for the shear test are about 850 mN, and the shear strength under this condition is 89.5 MPa. This also indicate that it is beneficial to improve the surface morphology and coating quality through grain refinement of the W-Cu alloys.

## 4. Conclusions

In this paper, two ultrafine-grained W-Cu alloys and two coarse-grained W-Cu alloys were prepared. The effects of different microstructures of W-Cu alloys on the surface morphology and dynamic mechanical properties of the W-Cu alloy under impact load were studied. It is found that in the coarse-grained W-Cu alloys, it is easy to form pits and cracks in the tungsten phase on the surface during impact loading, while the fine-grained tungsten alloy is not prone to this phenomenon. The simulation results show that refining the tungsten grains can not only reduce the impact absorption energy, but also increase the contact area of the tungsten and copper phases, thereby reducing the fracture probability of tungsten phase. The shear strength of gold studs on the Au/Ni coating is increased by about 33% after grain refinement for the W-Cu alloys. As a result, grain refinement is beneficial for improving the surface morphology and dynamic mechanical properties of the W-Cu alloys under impact loading.

## Figures and Tables

**Table 1:** The Johnson–Cook model constants.

Material	A (MPa)	B (MPa)	n	C	m
Tungsten (W) [26]	1200	1030	0.019	0.034	0.4
Copper (Cu) [26]	107	213	0.26	0.024	1.09

**Table 2:** The contact relations between the W-Cu alloys and alumina particle.

Contact Relations	CG WCu20	RD-CG WCu20	UFG WCu20
δ (Simulation result>	4.26 μm	9.64 μm	1.94 μm
δ/r′ (Calculation	0.045	0.107	0.020

## Data Availability

Data available in a publicly accessible repository.
